# The relationship between genotype and phenotype in Chinese children with glucose transporter type 1 deficiency syndrome

**DOI:** 10.3389/fneur.2025.1638549

**Published:** 2025-09-16

**Authors:** Mei-Jiao Zhang, Shi-Min Zhang, Qing-Ping Zhang, Yong-Xin Wen, Jia-Ping Wang, Hui Xiong, Yu-Wu Jiang, Xin-Hua Bao

**Affiliations:** ^1^Department of Pediatrics, Peking University First Hospital, Beijing, China; ^2^Department of Pediatrics, Peking University People’s Hospital, Beijing, China; ^3^Department of Pediatric Neurology, Guangdong Women and Children Hospital, Guangdong, China; ^4^Department of Neurology, Beijing Children’s Hospital, Capital Medical University, Beijing, China

**Keywords:** glucose transporter type 1 deficiency syndrome, *SLC2A1*, genotype-phenotype correlation, seizures, movement disorders

## Abstract

**Background:**

Glucose transporter type 1 deficiency syndrome (Glut1DS) is a treatable neurogenetic metabolic disorder caused by pathogenic variants in the *SLC2A1* gene. The relationship between genotype and phenotype has not been extensively studied in large cohorts within China. This study aimed to analyze the clinical and genetic characteristics and the genotype-phenotype correlation in Chinese children with Glut1DS.

**Methods:**

Clinical data of Glut1DS patients, including age of onset, clinical manifestations, cerebrospinal fluid (CSF) analysis, and *SLC2A1* gene variants, were collected and analyzed.

**Results:**

A total of 93 patients with Glut1DS were included, among whom 65 (70%) were classical phenotypes (including 55 early-onset classical cases and 10 late-onset classical cases), and 28 (30%) were non-classical phenotypes. Significant differences were observed among early-onset classical, late-onset classical, and non-classical groups in terms of age of onset (*p* < 0.001), episodic psychiatric/behavioral abnormalities (*p* = 0.012), CSF glucose levels (*p* < 0.001), and the ratio of CSF glucose to blood glucose (*p* = 0.003). Genetic variant analysis identified 40 previously reported and 32 novel *SLC2A1* variants. These variants were classified into three types: Type A (missense and in-frame indel variants, *n* = 52), Type B (frameshift, nonsense, splicing site, and initiation codon variants, *n* = 32), and Type C (single/multiple exon or whole-gene deletions, *n* = 9). No statistically significant difference was found in the distribution of these three genotypes across early-onset classical, late-onset classical, and non-classical phenotype. However, there were differences in age of onset among the three genetic variant groups (*p* = 0.009), with Type A variants showing a later age of onset compared to Type B variants (*p* = 0.014). No significant differences were observed among the three variant groups regarding CSF glucose levels, the ratio of CSF glucose to blood glucose, or CSF lactate levels. Furthermore, patients with identical variants exhibited phenotypic variability, for example, among eight patients (9%) harboring the c.997C>T (p.Arg333Trp) variant, six had early-onset classical phenotypes, while two had non-classical phenotypes.

**Conclusion:**

Glut1DS predominantly manifests as the classical phenotype, with the early-onset classical phenotype presenting at the youngest age and exhibiting the most severe clinical symptoms. Patients with this type also showed lower CSF glucose levels and a lower CSF glucose to blood glucose ratio compared to other types. Missense and in-frame indel variants were associated with a later age of onset compared to other types of genetic variants. No significant correlations were found between genotype and clinical classification or CSF glucose levels.

## Introduction

Glucose transporter type 1 (Glut1) protein is a critical carrier for glucose transport across the blood–brain barrier into neurons and glial cells. Pathogenic variants in the SLC2A1 gene lead to reduced Glut1 protein expression or functional impairment, resulting in Glucose transporter type 1 deficiency syndrome (Glut1DS), which was first described by De Vivo et al. (1). The first case of Glut1DS in China was diagnosed at Peking University First Hospital in 2008 and reported in 2012 (2, 3). The estimated incidence of Glut1DS in European and American populations ranges from 1.65 to 4.13 per 100,000 live births (4, 5). Clinically, Glut1DS can be categorized into classical phenotype (early-onset and late-onset) and non-classical phenotype. Classical phenotypes are primarily characterized by infantile refractory epilepsy, developmental delay, and complex movement disorders including spasticity, ataxia, and dystonia. Non-classical phenotypes include paroxysmal exertion-induced dyskinesias (PED), complex movement disorders, and intellectual disability. Due to its rarity, large-scale studies on the genotype-phenotype relationship in Chinese children with Glut1DS are currently lacking (6). This study analyzed data from 93 Glut1DS pediatric patients to summarize their phenotypic and genotypic characteristics and explore the correlation between genotype and phenotype.

## Methods

### Study population and inclusion criteria

Patients diagnosed with Glut1DS at Peking University First Hospital from November 2008 to January 2025 were included in this study.

Diagnostic criteria were based on the 2025 diagnosis and treatment recommendations (7): (1) clinical manifestations, such as seizures, episodic or persistent movement disorders triggered by fasting, fatigue, or physical activity, and psychomotor developmental delays; (2) low CSF glucose levels (<2.8 mmol/L) with a CSF to blood glucose ratio <0.6; and (3) pathogenic *SLC2A1* variants.

Exclusion criteria included (1) increased CSF white blood cell count, protein, or lactate levels; (2) other conditions causing reduced CSF glucose; (3) *SLC2A1* variants classified as benign or likely benign according to the American College of Medical Genetics and Genomics (ACMG) guidelines.

Inclusion criteria required patients fulfill the diagnostic criteria (1), (2), and (3), or diagnostic criteria (1) and (3), as well as patients with negative or absent *SLC2A1* testing, diagnostic criteria (1) and (2) were met and all exclusion criterion satisfied.

Clinical classifications were defined as follows: (1) Classical phenotype: primarily characterized by epilepsy, further subdivided into: ① Early-onset classical phenotype: epilepsy onset before age 2 years. ② Late-onset classical phenotype: epilepsy onset after age 2 years. (2) Non-classical phenotype: primarily characterized by movement disorders and/or developmental delay, with or without epilepsy.

Gene classification was based on the potential impact of variants on protein function, ranked from mild to severe: Type A: missense variants and in-frame indel variants; Type B: frameshift, nonsense, splicing site, and initiation codon variants; Type C: single, multiple exon, or whole-gene deletions.

This study was approved by the Clinical Research Ethics Committee of Peking University First Hospital (Approval No. 2021 Research 332), and informed consent was obtained from all parents.

### Data collection and methods

Clinical data were collected for each patient, including: (1) Basic information: age, gender, etc. (2) Clinical data: age of onset, initial symptoms, psychomotor developmental status, presence of epilepsy or movement disorders, etc. (3) Laboratory tests: CSF analysis, concurrent blood glucose, genetic testing, etc.

Lumbar puncture for CSF collection was performed after a 4–6-h fast, with pre-puncture blood glucose measurement. CSF tests included routine, biochemical, and lactate analyses.

Peripheral blood samples were collected from patients and their parents, and genomic DNA was extracted following standard procedures. Prior to 2013, polymerase chain reaction (PCR) combined with Sanger sequencing was used for *SLC2A1* gene mutational test; after 2013, next-generation sequencing (NGS) was employed. Family members of patients with identified *SLC2A1* variants were verified. If no definitive pathogenic variant was detected, multiplex ligation-dependent probe amplification (MLPA) or low-depth whole-genome copy number variation sequencing (CNV-Seq) was used to screen for large deletions or duplications in the *SLC2A1* gene. Variant pathogenicity was assessed according to the guidelines of the American College of Medical Genetics and Genomics (ACMG).

### Data analysis

Data were analyzed using SPSS 26.0. Categorical variables are presented as counts (%), categorical data are presented as number of cases (%). Normally distributed continuous data are expressed as range (mean ± SD), and non-normally distributed continuous data are expressed as range (median). Continuous variable comparisons were performed using rank-sum tests, and categorical data were analyzed using chi-square tests or Fisher’s exact tests. A *p*-value <0.05 indicated statistical significance.

## Results

### Basic characteristics

A total of 93 patients with Glut1DS were included in this study. Among them, 72 patients (77.4%) met all three diagnostic criteria, 17 patients (18.3%) met diagnostic criteria 1 and 3, confirming a diagnosis of Glut1DS, and 4 patients (4.3%) had *SLC2A1* gene variants classified as uncertain significance by ACMG guidelines but fulfilled diagnostic criteria 1 and 2 along with all exclusion criteria, leading to a clinical diagnosis of Glut1DS. There were 51 males (55%) and 42 females (45%). The age of onset ranged from 1 month to 168 months (median 8 months), and the age at diagnosis ranged from 2 months to 228 months (median 43 months). At the last follow-up in January 2025, patient ages ranged from 1.3 years to 27.8 years (median 10.5 years), with disease durations ranging from 0.7 years to 13.8 years (median 9.1 years).

### Clinical features

Of the 93 Glut1DS patients, 65 (70%) were classic phenotype, including 55 early-onset classic cases and 10 late-onset classic cases, while 28 (30%) were non-classic phenotype. The age of onset for classic cases ranged from 1 to 144 months (median 4 months), with early-onset classic cases presenting between 1 and 22 months (median 3 months) and late-onset classic cases presenting between 24 and 144 months (median 37 months). Non-classic cases presented between 3 and 168 months (median 15 months). The age of onset was significantly younger in classic cases compared to non-classic cases (*p* < 0.001).

Seventy-one patients (76%, 71/93) experienced epileptic seizures, with the first seizure occurring between 1 and 144 months (median 25 months). Among these, 45 patients exhibited a single seizure type, while 26 patients had multiple seizure types. The seizure types included generalized tonic-clonic seizures (19 cases), myoclonic seizures (11 cases), absence seizures (9 cases) including six cases of early-onset absence epilepsy with ages under 4 years, atonic seizures (5 cases), generalized tonic seizures (4 cases), myoclonic-atonic seizures (3 cases), eyelid myoclonia (2 cases), and focal seizures (49 cases). Five patients experienced status epilepticus.

Seventeen patients (18%, 17/93) exhibited episodic eye-head movements characterized by rapid, multidirectional eye movements often accompanied by head movements in the same direction. These movements were first observed between 2 months and 1.5 years (median 4 months), with 12 cases (71%) occurring within the first 6 months. It disappeared between 1 year and 4 years (median 2.5 years). Fifteen of these patients also had epilepsy. The eye-head movements presented before the onset of epilepsy in 10 cases (67%), and after epilepsy onset in 5 cases.

Fifty-nine patients (63%, 59/93) experienced movement disorders, which appeared between 1 year and 14 years (median 2.3 years). The most common movement disorder was ataxia (29 cases), followed by ataxia-spastic gait (13 cases), dystonia (9 cases), and PED (8 cases). Seventeen patients had persistent movement disorders without episodic exacerbation, including ataxia (5 cases), ataxia-spastic gait (8 cases), and dystonia (4 cases). Twenty-two patients had persistent movement disorders with episodic exacerbation, including ataxia (15 cases), ataxia-spastic gait (5 cases), and dystonia (2 cases). Twenty patients had episodic movement disorders, including ataxia (9 cases), PED (8 cases), and choreoathetosis (3 cases), lasting between 20 min and 2 h. Long-distance walking was a common trigger for PED, while fasting was the most frequent trigger for ataxia and dystonia, followed by fatigue, infection, and emotional stress.

Movement disorders were present in 56% (31/55) of early-onset classic cases, 50% (5/10) of late-onset classic cases, and 82% (23/28) of non-classic cases, with significant differences among groups (*p* = 0.045). Specifically, the incidence of movement disorders was significantly higher in non-classic cases compared to early-onset classic cases (*p* = 0.020). No significant differences were found between early-onset and late-onset classic cases (*p* = 0.979) or between late-onset classic and non-classic cases (*p* = 0.090). Episodic psychiatric and behavioral abnormalities were observed in 9% (5/55) of early-onset classic cases, 20% (2/10) of late-onset classic cases, and 36% (10/28) of non-classic cases, with a significant difference noted between early-onset classic and non-classic cases (*p* = 0.022).

**Figure 1:**
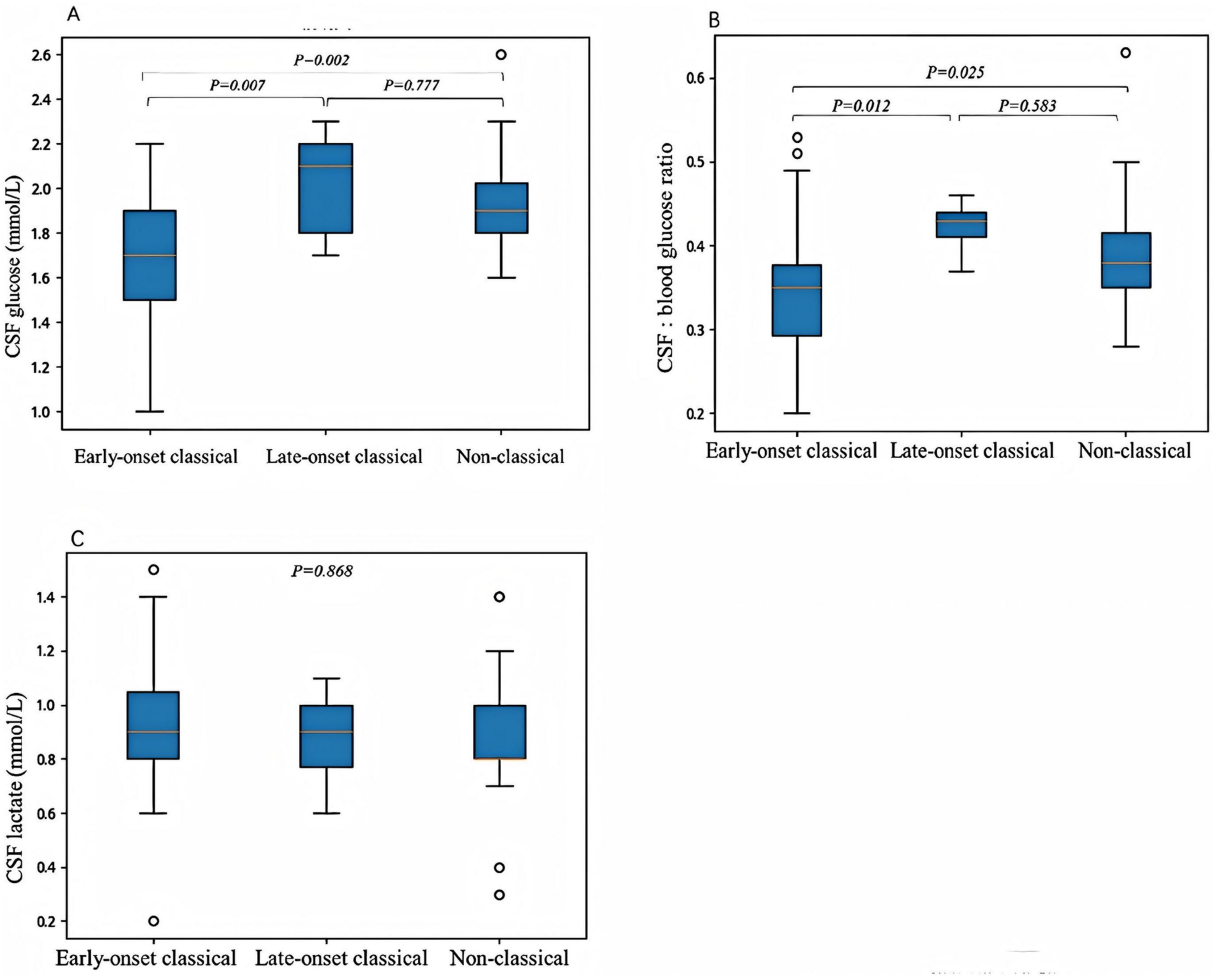
The correlations between clinical phenotypes and cerebrospinal fluid (CSF) glucose levels **(A)**, CSF to blood glucose ratios **(B)**, and CSF lactate levels **(C)**. **(A)** The CSF glucose levels were significantly lower in the early-onset classic type compared to the late-onset classic type (*p* = 0.007) and the non-classic type (*p* = 0.002). No significant difference was observed between the late-onset classic and non-classic subtypes (*p* = 0.777). **(B)** The CSF to blood glucose ratios were significantly lower in the early-onset classic type compared to the late-onset classic type (*p* = 0.012) and the non-classic type (*p* = 0.025). There was no significant difference in the CSF to blood glucose ratios between the late-onset classic and non-classic types (*p* = 0.583). **(C)** No significant differences were observed in CSF lactate levels among the three subtypes (*p* = 0.868). Differences were considered statistically significant at *p* < 0.05.

Cerebrospinal fluid (CSF) glucose levels were recorded in 76 patients (82%, 76/93), ranging from 1 to 2.6 mmol/L (median 1.8 mmol/L), with 69 patients (91%, 69/76) having CSF glucose levels below 2.2 mmol/L. Blood glucose was measured concurrently in 74 patients (80%, 74/93), with the CSF to blood glucose ratio ranging from 0.20 to 0.63 (median 0.37), and 65 patients (88%, 65/74) having ratios below 0.45. CSF lactate was measured in 41 patients (44%, 41/93), with values ranging from 0.2 to 1.5 mmol/L (median 0.9 mmol/L). All patients had normal CSF cell counts. CSF glucose levels were 1–2.2 mmol/L (median 1.7 mmol/L) in early-onset classic cases, 1.7–2.3 mmol/L (median 2.0 mmol/L) in late-onset classic cases, and 1.6–2.6 mmol/L (median 1.9 mmol/L) in non-classic cases. Early-onset classic cases had significantly lower CSF glucose levels compared to late-onset classic (*p* = 0.007) and non-classic cases (*p* = 0.002), with no significant difference between late-onset classic and non-classic cases (*p* = 0.777). The CSF to blood glucose ratio was 0.22–0.53 (median 0.34) in early-onset classic cases, 0.37–0.46 (median 0.42) in late-onset classic cases, and 0.28–0.63 (median 0.39) in non-classic cases. Early-onset classic cases had significantly lower ratios compared to late-onset classic (*p* = 0.012) and non-classic cases (*p* = 0.025), with no significant difference between late-onset classic and non-classic cases (*p* = 0.583). The CSF lactate levels in the early-onset classic type was 0.2–1.5 mmol/L (median 0.93 mmol/L), in the late-onset classic type was 0.6–1.1 mmol/L (median 0.87 mmol/L), and in the non-classic type was 0.3–1.4 mmol/L (median 0.88 mmol/L). CSF lactate levels did not differ significantly among the three groups (*p* = 0.868) (Figure 1).

In this cohort, CSF glucose levels in early-onset absence epilepsy patients were 1.8–2.0 mmol/L (median 1.8 mmol/L), with a CSF to blood glucose ratio of 0.37–0.46 (median 0.38). In patients with absence seizures aged over 4 years, CSF glucose levels were 1.2–2.2 mmol/L (median 1.8 mmol/L), with a CSF to blood glucose ratio of 0.23–0.46 (median 0.45). In patients with PED, CSF glucose levels were 1.6–2.0 mmol/L (median 2.2 mmol/L), with a CSF to blood glucose ratio of 0.28–0.40 (median 0.36). Due to the small sample size, no statistical analysis according to the symptoms was performed.

### Features of genetic variations

Seventy-two distinct *SLC2A1* gene variants were found in 93 patients with Glut1DS. These were categorized into three types: Type A variants (52 cases), which included missense mutations (48 cases) and in-frame indel mutations (4 cases); Type B variants (32 cases), which consisted of frameshift mutations (19 cases), nonsense mutations (7 cases), splicing mutations (4 cases), and initiation codon mutations (2 cases); and Type C variants (9 cases), which included single-exon deletions (1 case), multi-exon deletions (5 cases), and whole-gene deletions (3 cases). Specifically, Type A variants were observed in 28 early-onset classic cases, 9 late-onset classic cases, and 15 non-classic cases. Type B variants were found in 21 early-onset classic cases, 1 late-onset classic case, and 10 non-classic cases. Type C variants included 1 non-classic case with a single-exon deletion, 4 early-onset classic cases and 1 non-classic case with multi-exon deletions, and 2 early-onset classic cases and 1 non-classic case with whole-gene deletions.

The most common mutation was c.997C>T (p.Arg333Trp), identified in 8 patients (9%, 8/93). Other frequent mutations included c.988C>T (p.Arg330*), c.274C>T (p.Arg92Trp), and c.376C>T (p.Arg126Cys), each detected in 6, 3, and 3 patients, respectively. The mutations c.102T>A (p.Asn34Lys), c.398G>A (p.Cys133Tyr), c.457C>T (p.Arg153Cys), c.2T>A (p.Met1?), and c.680-1G>A were each observed in 2 patients. The remaining 63 variants were identified in only 1 patient each.

### Relationship between genotype and phenotype

**Figure 2:**
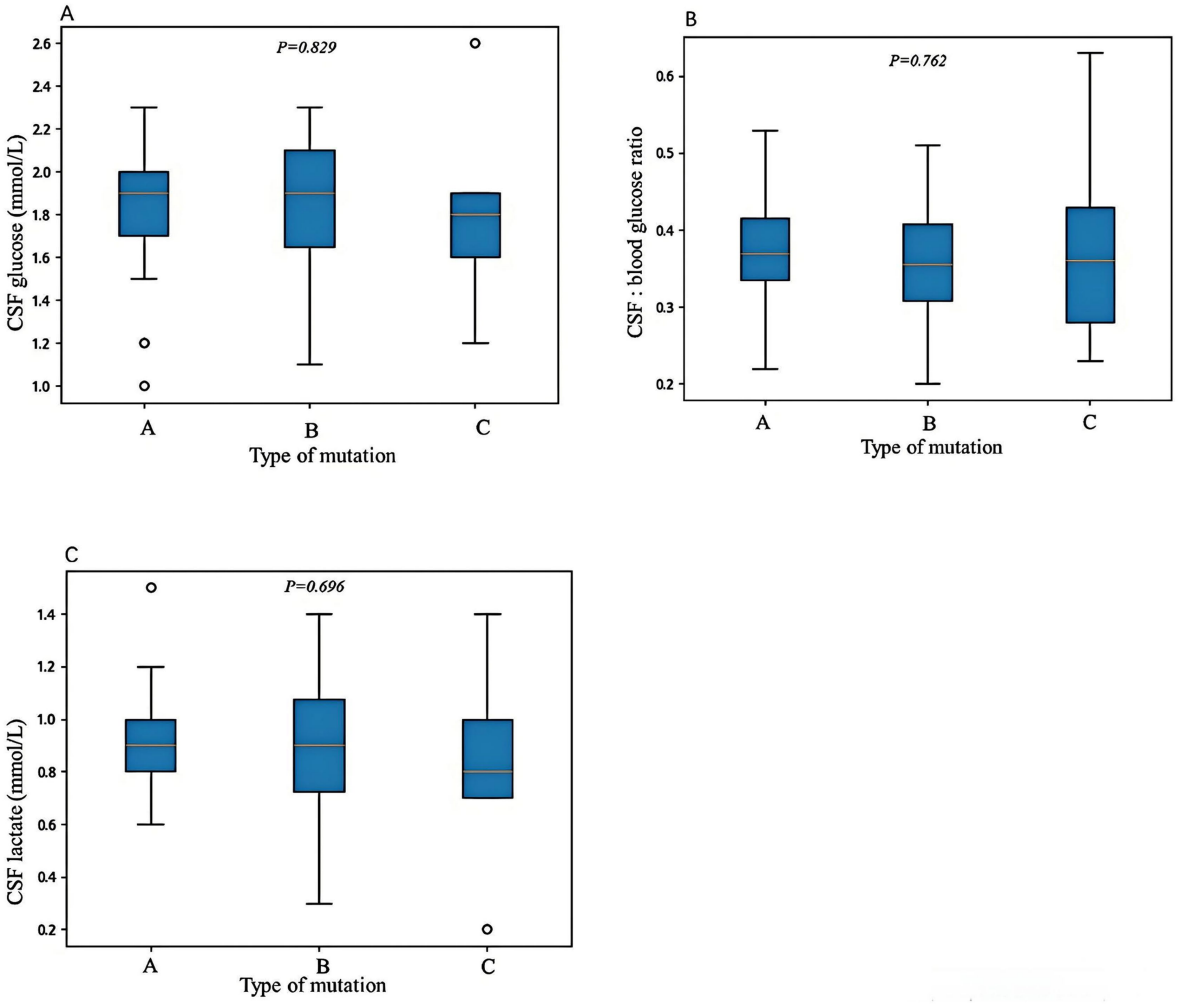
The correlations between genetic variant types and cerebrospinal fluid (CSF) glucose levels **(A)**, CSF to blood glucose ratios **(B)**, and CSF lactate levels **(C)**. Variant Type A includes missense variants and in-frame indel variants; variant Type B includes frameshift, nonsense, splicing, and initiation codon variants; and variant Type C includes single-exon, multi-exon, or whole-gene deletions. No statistically significant differences were observed among the three variant types in terms of CSF glucose levels, CSF to blood glucose ratios, or CSF lactate levels. Differences were considered statistically significant at ^*^p < 0.05.

The age of onset for Type A variants ranged from 1 to 168 months (median 12 months), for Type B variants from 1 to 144 months (median 4 months), and for Type C variants from 1.4 to 37 months (median 5 months). Statistical analysis revealed that the age of onset was significantly older in Type A compared to Type B variants (*p* = 0.014), while no significant differences were observed between Type A and Type C (*p* = 0.141) or Type B and Type C (*p* = 1.000). There was no statistically significant difference in the distribution of genetic variant types among the three clinical phenotypes (early-onset classic, late-onset classic, and non-classic; *p* = 0.298). In Type A variants, 65% (34/52) exhibited movement disorders, with cerebrospinal fluid (CSF) glucose levels ranging from 1 to 2.3 mmol/L (median 1.83 mmol/L), CSF to blood glucose ratios from 0.2 to 0.5 (median 0.37), and CSF lactate levels from 0.6 to 1.48 mmol/L (median 0.93 mmol/L). In Type B variants, movement disorders were present in 63% (20/32) of cases, with CSF glucose levels ranging from 1.1 to 2.3 mmol/L (median 1.8 mmol/L), CSF to blood glucose ratios from 0.2 to 0.5 (median 0.36), and CSF lactate levels from 0.3 to 1.4 mmol/L (median 0.9 mmol/L). In Type C variants, movement disorders occurred in 56% (5/9) of cases, with CSF glucose levels ranging from 1.2 to 2.6 mmol/L (median 1.77 mmol/L), CSF to blood glucose ratios from 0.23 to 0.63 (median 0.36), and CSF lactate levels from 0.2 to 1.4 mmol/L (median 0.82 mmol/L). No significant differences were observed in the incidence of movement disorders or CSF lactate levels among the three groups (*p* = 0.844 and *p* = 0.696, respectively). Although Type A variants showed slightly higher CSF glucose levels and CSF to blood glucose ratios, these differences were not statistically significant (*p* = 0.829 and *p* = 0.762, respectively) (Figure 2).

Based on the regions of Glut1 protein affected by the mutations, the variants were further classified into three groups: transmembrane domain (27 cases), intracellular loop (35 cases), and extracellular loop (18 cases). Variants in the transmembrane domain, the age of onset ranged from 1.1 to 88 months (median 8 months), with 44% (12/27) being early-onset classic, 22% (6/27) late-onset classic, and 34% (9/27) non-classic cases. Movement disorders were present in 56% (15/27) of cases, with CSF glucose levels ranging from 1 to 2.3 mmol/L (median 1.85 mmol/L), CSF to blood glucose ratios from 0.22 to 0.5 (median 0.38), and CSF lactate levels from 0.6 to 1.5 mmol/L (median 0.93 mmol/L). Type A variants accounted for 74% (20/27), and Type B variants for 26% (7/27), while Type C variants (single or multi-exon deletions or whole-gene deletions) were excluded from this classification. Variants in the intracellular loop, the age of onset ranged from 1 to 168 months (median 11 months), with 57% (20/35) being early-onset classic, 6% (2/35) late-onset classic, and 37% (13/35) non-classic cases. Movement disorders were present in 74% (26/35) of cases, with CSF glucose levels ranging from 1.1 to 2.3 mmol/L (median 1.81 mmol/L), CSF to blood glucose ratios from 0.21 to 0.53 (median 0.37), and CSF lactate levels from 0.8 to 1.4 mmol/L (median 0.93 mmol/L). Type A variants accounted for 57% (20/35), and Type B variants for 43% (15/35). Variants in the extracellular loop, the age of onset ranged from 8 to 168 months (median 4 months), with 78% (14/18) being early-onset classic, 11% (2/18) late-onset classic, and 11% (2/18) non-classic cases. Movement disorders were present in 56% (10/18) of cases, with CSF glucose levels ranging from 1.1 to 2.2 mmol/L (median 1.77 mmol/L), CSF to blood glucose ratios from 0.2 to 0.45 (median 0.35), and CSF lactate levels from 0.3 to 1.2 mmol/L (median 0.89 mmol/L). Type A and Type B variants each accounted for 50% (9/18). No significant differences were observed among the three groups in terms of age of onset (*p* = 0.117), clinical phenotypes (*p* = 0.078), incidence of movement disorders (*p* = 0.224), CSF glucose levels (*p* = 0.736), CSF to blood glucose ratios (*p* = 0.292), CSF lactate levels (*p* = 0.930), or genetic variant types (*p* = 0.222).

## Discussion

Glut1DS exhibits a wide range of phenotypic variability, classified based on clinical manifestations and age of onset into classic types (including early-onset classic and late-onset classic) and non-classic types. Among these, the early-onset classic type is the most severe and has the earliest age of onset. In this cohort, the early-onset classic type presented between 1 and 22 months (median 3 months), the late-onset classic type between 24 and 144 months (median 37 months), and the non-classic type between 3 and 168 months (median 15 months). The classic type had a significantly younger age of onset compared to the non-classic subtype (*p* < 0.001).

It was suggested that the severity of Glut1DS phenotypes correlates with the residual function of the GLUT1 protein, which is responsible for glucose transport from blood into the brain and red blood cells. Therefore, CSF glucose levels, particularly the CSF to blood glucose ratio, as well as red blood cell glucose uptake rates in patients with Glut1DS, may reflect the residual function of the GLUT1 protein to some extent. Previous studies have reported that higher CSF to blood glucose ratios are associated with milder Glut1DS phenotypes, while lower ratios correlate with more severe phenotypes (8–11). Nabatame et al. (12) recently found that higher CSF glucose levels in Glut1DS patients were associated with better neurological development. However, other report indicate that the timing of symptom onset is unrelated to red blood cell glucose uptake rates (13). In this cohort, both the CSF glucose levels and the CSF to blood glucose ratios were lower in the early-onset classic type compared to the late-onset classic and non-classic types, supporting the notion that patients with lower CSF glucose levels and CSF to blood glucose ratios tend to present earlier and exhibit more severe clinical symptoms. This confirms that the degree of GLUT1 protein dysfunction caused by SLC2A1 gene variants correlates with the severity of the clinical phenotype. Leen et al. (14) also demonstrated that the CSF to blood glucose ratio was lower in the early-onset classic type compared to the late-onset classic (*p* = 0.009) and non-classic types (*p* = 0.005), although no significant differences were observed in absolute CSF glucose levels among the three types. This discrepancy may be explained by the fact that CSF glucose levels reflect only a single time point and are influenced by concurrent blood glucose levels, whereas the CSF to blood glucose ratio provides a better reflection of GLUT1 protein transport function.

The relationship between residual GLUT1 protein function and clinical symptoms remains unclear. Several studies using the Xenopus oocyte expression system have investigated the relationship between different clinical symptoms and corresponding gene variants in terms of glucose uptake rates (15–18). Variants associated with early-onset absence epilepsy exhibited lower glucose uptake rates compared to those causing childhood absence epilepsy, and variants linked to PED showed even lower glucose uptake rates. Thus, PED appears to represent the most severe impairment of GLUT1 function, followed by early-onset absence epilepsy and typical absence epilepsy. In this cohort, the median CSF glucose level in early-onset absence epilepsy patients was 1.8 mmol/L, with a median CSF to blood glucose ratio of 0.38; in childhood absence epilepsy patients, the median CSF glucose level was also 1.8 mmol/L, but the median CSF to blood glucose ratio was 0.45; in PED patients, the median CSF glucose level was 2.2 mmol/L, with a median CSF to blood glucose ratio of 0.36. These findings suggest that the CSF to blood glucose ratio in early-onset absence epilepsy patients is lower than in childhood absence epilepsy patients, and even lower in PED patients, consistent with trends in the above studies. However, due to the small sample size, no statistical analysis was performed. Notably, the median CSF glucose level in PED patients was higher than in early-onset and childhood absence epilepsy patients, indicating that CSF glucose levels and the CSF to blood glucose ratio do not always correlate perfectly.

Though there is a certain correlation between residual GLUT1 function and Glut1DS phenotypes, the impact of different SLC2A1 gene variants on GLUT1 function and the relationship between genotype and phenotype remain unclear. Wang et al. (19) speculated in 2005 that large deletions, nonsense mutations, frameshift mutations, and splice site mutations significantly impair GLUT1 protein function, leaving approximately 50% residual function and causing moderate phenotypes. Some missense variants result in approximately 50–75% residual GLUT1 function, leading to mild phenotypes characterized by mild motor disorders, while other missense variants preserve more than 75% residual function, causing the mildest phenotypes or asymptomatic cases, with symptoms appearing only under specific triggers such as fatigue, fever, or fasting. Similarly, Yang et al. (20) found that missense variants predominantly occur in patients with mild and moderate phenotypes, while splice site, nonsense, insertion, deletion, and multi-exon deletion variants almost exclusively occur in patients with moderate to severe phenotypes, and complete gene deletions are associated with severe phenotypes. In order to better analyze the relationship between genotype and phenotype, Leen et al. (14) categorized the genotypes of 57 Glut1DS patients into three groups: Group A (missense variants), Group B (frameshift, nonsense, splice site, and initiation codon variants), and Group C (multi-exon deletions). Their analysis revealed that patients with Group B and Group C variants had significantly lower CSF glucose levels and CSF to blood glucose ratios compared to those with Group A variants. Mauri et al. (21) also found that the CSF to blood glucose ratio was significantly lower in Group B patients compared to Group A patients. These findings indicate that missense variants have a lesser impact on GLUT1 function, while frameshift, nonsense, splice site, initiation codon, and multi-exon deletion variants significantly impair GLUT1 function. However, in this cohort study, despite a similar grouping strategy, no statistically significant differences were observed in CSF glucose levels or CSF to blood glucose ratios among the three variant types, suggesting no definitive correlation between genotype and residual GLUT1 function. This discrepancy may be attributed to differences in genetic backgrounds across populations or the influence of modifier genes. Future studies with larger sample sizes and cross-population analyses are needed to clarify the effects of different variant types on GLUT1 function.

In studies of the relationship between genotype and phenotype, Leen et al. (14) found that 65% of individuals with Type A variants exhibited the early-onset classical phenotype, compared to 62% for Type B variants and 100% for Type C variants. No statistically significant differences were observed in the distribution of phenotypes among the three variant groups. Similarly, no significant differences were observed in this cohort. Leen et al. (14) further demonstrated that patients with Group B or Group C variants were more likely to develop movement disorders compared to those with Group A variants (*p* = 0.037). Additionally, the severity of developmental delay correlated with variant type, with Group A patients exhibiting predominantly mild developmental delays compared to Groups B and C (*p* < 0.001). In this cohort, no statistically significant differences in movement disorders were observed among the three variant types. Due to the different developmental assessment methods used in this study, no correlation analysis was performed between variant type and the severity of developmental delay. Akman et al. (13) reported that the timing of symptom onset was unrelated to variant type. The earlier onset was associated with a higher likelihood of episodic eye-head movements and lower Columbia Neurological Scores (CNS) (22). In this cohort, 88% of patients with episodic eye-head movements exhibited classic phenotypes and had an earlier age of onset. However, patients with Group A variants had a significantly later age of onset compared to those with Group B variants (*p* = 0.013), with no significant differences between Groups A and C (*p* = 0.141) or Groups B and C (*p* = 1.000). Anyway, the results from previous studies are inconsistence, indicated no definitive correlation between Glut1DS phenotype and variant type. Furthermore, multiple studies have reported phenotypic heterogeneity among patients with identical variants (21, 23–25). In this cohort, eight patients had the same variant, c.997C>T (p.Arg333Trp), with six exhibiting early-onset classic phenotypes and two non-classic phenotypes. This phenotypic diversity suggests that the relationship between genotype and phenotype is not definitive. Other genetic or environmental factors may play important roles in disease manifestation.

Based on the regions of GLUT1 protein affected by the mutations, they were divided into transmembrane domain, intracellular loop, and extracellular loop. No significant differences were observed among the three groups in terms of age of onset, CSF glucose levels, CSF to blood glucose ratios, CSF lactate levels, or variant types, indicating that all regions of the GLUT1 protein are critical for its function.

In summary, Glut1DS is a treatable neurogenetic metabolic disorder caused by SLC2A1 gene variants. The severity of clinical phenotypes correlates with the degree of GLUT1 protein dysfunction caused by SLC2A1 variants. Patients with the early-onset classic phenotype exhibit the lowest CSF glucose levels and CSF to blood glucose ratios and the most severe clinical symptoms. Missense and in-frame variants are associated with a later age of onset, but no statistically significant differences were observed between clinical phenotypes, CSF glucose levels, CSF to blood glucose ratios, and genotype. Phenotypic variability among patients with identical gene variants suggests that other genes or regulatory proteins may also play roles in glucose transportation.

## Data Availability

The raw data supporting the conclusions of this article will be made available by the authors, without undue reservation.
